# Methimazole‐induced ANCA‐associated vasculitis with diffuse alveolar haemorrhage

**DOI:** 10.1002/rcr2.315

**Published:** 2018-05-08

**Authors:** Naoki Arai, Kenji Nemoto, Shuji Oh‐ishi, Mizu Nonaka, Kenji Hayashihara, Takefumi Saito

**Affiliations:** ^1^ National Hospital Organization Ibarakihigashi National Hospital Naka‐gun Ibaraki Japan

**Keywords:** Antineutrophil cytoplasmic antibody, methimazole, vasculitis, alveolar haemorrhage

## Abstract

Antineutrophil cytoplasmic antibody (ANCA)‐associated vasculitis (AAV) caused by methimazole (MMI) is known to be relatively rare; therefore, the optimal therapeutic approach for these cases remains to be established. A 59‐year‐old man who was treated with MMI for a diagnosis of Graves’ disease was referred to our hospital because of progressive haemoptysis. The patient was diagnosed with diffuse alveolar haemorrhage (DAH) secondary to AAV based on increased inflammatory reactions with positive myeloperoxidase‐ANCA in the serum and the results of bronchoalveolar lavage fluid. MMI was suspected as the cause of the AAV; therefore, the administration of MMI was discontinued. Thereafter, the patient’s symptoms as well as chest radiographic abnormalities completely resolved, in conjunction with normalization of the serum ANCA level. Our experience with this case suggests that DAH secondary to AAV caused by MMI may improve with discontinuation of the offending drug alone, with no other treatment.

## Introduction

Antineutrophil cytoplasmic antibody (ANCA)‐associated vasculitis (AAV) caused by antithyroid drugs is known to be relatively common, and in most cases, the culprit drug is propylthiouracil (PTU) 1. Therefore, little information on the optimal treatment of AAV caused by methimazole (MMI) is available. Indeed, limited data on diffuse alveolar haemorrhage (DAH) secondary to AAV caused by MMI suggested that the addition of steroid therapy is associated with an improvement of clinical conditions 2, 3. Here, we present a rare case of MMI‐induced AAV with DAH, which improved with discontinuation of MMI alone, without any additional treatment.

## Case Report

**Figure 1:**
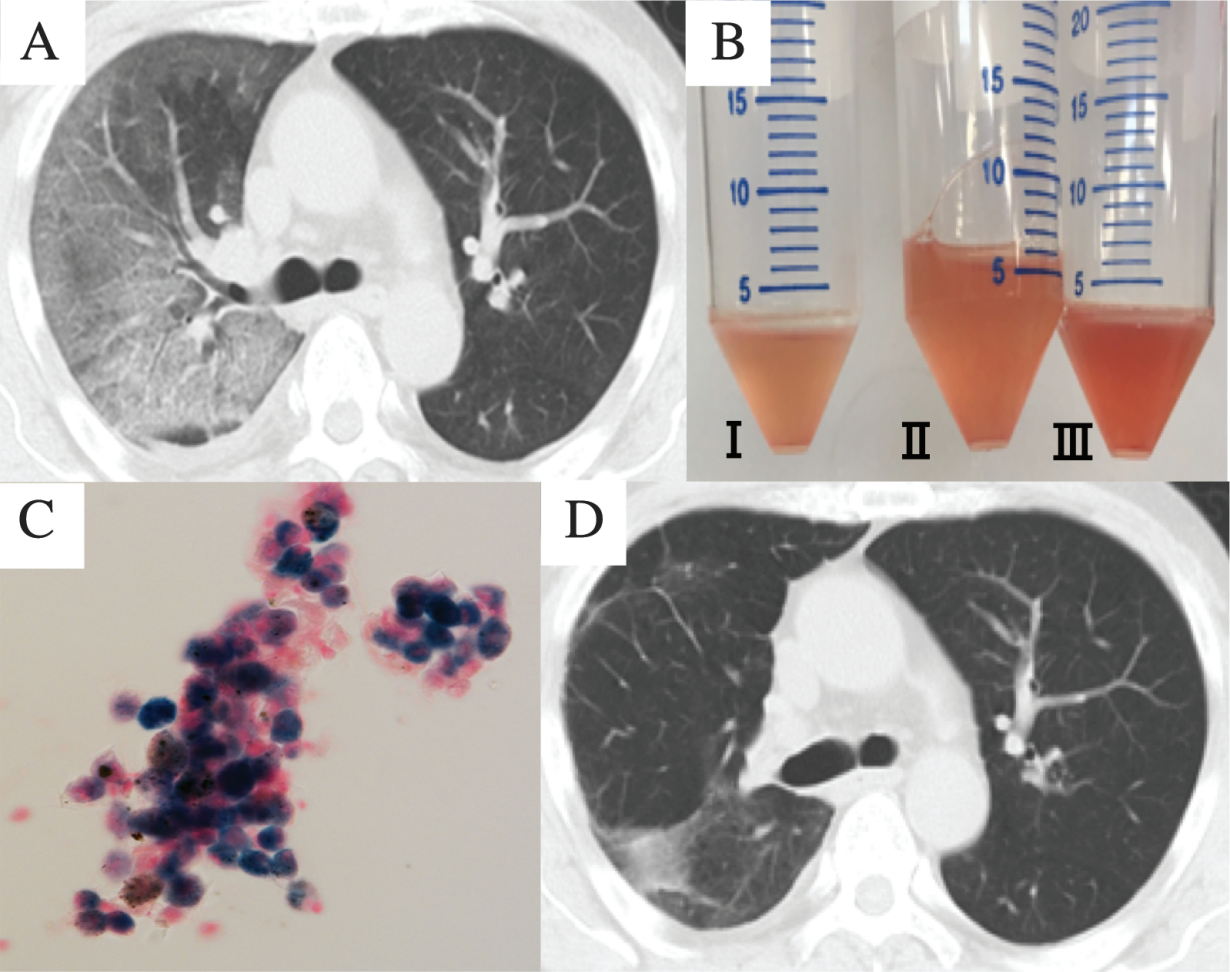
(A) Chest computed tomographic image showing a diffuse ground‐glass opacity in the right upper and middle lobes. (B) The bronchoalveolar lavage fluid (BALF) was haemorrhagic, and a concentration gradient was observed from I to III. (C) Cytological examination of the BALF revealed hemosiderin‐laden macrophages (Prussian blue, 400×). (D) Chest computed tomographic showing complete resolution of the diffuse ground‐glass opacity following discontinuation of methimazole (MMI).

A 59‐year‐old man who had been diagnosed with Graves’ disease in 2001 was started on treatment with MMI 5–10 mg/daily in 2009. In 2017, he was referred to our hospital because of progressive haemoptysis. On admission, his temperature was 38.1°C. Arterial blood gas analysis on room air showed a pH of 7.452, partial pressure of carbon dioxide (PaCO_2_) 34.0 mmHg, partial pressure of oxygen (PaO_2_) 92.8 mmHg, and bicarbonate (HCO_3_) 23.2 mmol/L. Chest computed tomography (Fig. 1A) showed a diffuse ground‐glass opacity in the right upper and middle lobes. Laboratory findings demonstrated increases in white blood cell count (12,600/μL) and serum C‐reactive protein (CRP, 17.8 mg/dL) and decrease of the haemoglobin (10.9 g/dL). The blood urea nitrogen concentration was 23.7 mg/dL, and the serum creatinine level was 2.42 mg/dL. Urinalysis revealed microhematuria and proteinuria. The serum‐free T4 and thyroid stimulating hormone (TSH) levels were 0.22 ng/mL and 15.89 μIU/mL, respectively, suggestive of hypothyroidism caused by MMI. The myeloperoxidase (MPO)‐ANCA level was elevated to 22.6 IU/mL (normal range <3.5 IU/mL), while the serum levels of other autoantibodies, such as proteinase‐3‐ANCA and anti‐nuclear antibodies, were within normal limits. The bronchoalveolar lavage fluid (BALF) demonstrated total cell 7.0 × 10^5^/mL, alveolar macrophages 81%, lymphocytes 3%, neutrophils 16%, eosinophil 0%, and haemorrhages (Fig. 1B) and showed abundant hemosiderin‐laden macrophages (Fig. 1C) with no bacteria, suggestive of DAH. Pathological findings of lung biopsy did not reveal a definitive diagnosis; however, other causes of DAH, such as systemic lupus erythematosus, goodpasture syndrome, and abnormal coagulation, seemed to be negative. Therefore, the patient was diagnosed as having DAH secondary to AAV. MMI was suspected as the cause of the AAV and discontinued. Thereafter, the patient’s symptoms as well as chest radiographic abnormalities completely resolved (Fig. 1D), in conjunction with normalization of the serum CRP, renal function parameters, and serum MPO‐ANCA level. After one month of MMI discontinuation, renal biopsies was performed; however, it was not related to histological features of ANCA‐related glomerulonephritis. Currently, 10 months after the discontinuation of MMI administration, the patient remains well, with no recurrence of DAH.

## Discussion

This patient developed DAH associated with AAV during MMI therapy. After discontinuation of MMI, all clinical abnormalities as well as the serum MPO‐ANCA level improved without any additional treatment. These results suggest that the AAV manifesting as DAH was indeed induced by MMI.

In the present case, AAV developed eight years after the initiation of MMI therapy, and the dose of MMI was low (5–10 mg/daily). With regard to these suspicions, Noh et al. 1 reported that AAV induced by antithyroid drugs could develop even after several years and occur even at low doses of these drugs. Therefore, the clinical course of this case seemed not to be incompatible with AAV induced by MMI.

In a previous case report of PTU‐induced AAV, pathological findings of renal biopsy were similar to that seen in non‐drug‐induced AAV 4. This case had abnormal renal function parameters consistent with ANCA‐related glomerulonephritis; however, renal biopsy did not reveal typical findings such as crecentic or necrotizing glomerulonephritis. One possible explanation is that the timing of the renal biopsy may be too late to obtain typical pathological findings. In fact, when renal biopsy was performed, the patient’s renal abnormalities had already improved.

Although the relationship between MMI and AAV has yet to be clearly established, Nakazawa et al. 5 reported that PTU‐induced AAV is associated with abnormalities of neutrophil extracellular traps (NETs). They showed that PTU induced abnormal conformation and impaired degradation of NETS 5. As the abnormal NETs led to the production of MPO‐ANCA and pulmonary capillaritis, Nakazawa et al. 5 speculated that PTU may play a certain role in the pathogenesis of AAV. Therefore, although further clinical studies are needed, a similar mechanism may also underlie MMI‐induced AAV.

The treatment of AAV caused by antithyroid drugs remains to be established. Until now, only two cases of MMI‐induced AAV with DAH have been reported, and both needed steroid treatment because of marked hypoxemia 2, 3. On the other hand, although DAH and renal dysfunction were detected, this case did not demonstrate dyspnoea with hypoxemia, and his general condition was relatively stable; therefore, steroid therapy was not used. To our knowledge, this is the first reported case in which MMI discontinuation alone, without any additional treatment, led to spontaneous remission of AAV.

In conclusion, AAV can be induced not only by PTU but also by MMI, and manifests as DAH. Our experience with this case also suggests that DAH secondary to AAV caused by MMI may resolve with discontinuation of the offending drug alone, with no other treatment.

### Disclosure Statement

Appropriate written informed consent was obtained for publication of this case report and accompanying images.
